# Thermal neuromodulation using pulsed and continuous infrared illumination in a penicillin-induced acute epilepsy model

**DOI:** 10.1038/s41598-023-41552-0

**Published:** 2023-09-02

**Authors:** Ebrahim Ismaiel, Richárd Fiáth, Ágnes Szabó, Ágoston Csaba Horváth, Zoltán Fekete

**Affiliations:** 1 Research Group for Implantable Microsystems, Faculty of Information Technology and Bionics, Pázmány Péter Catholic University, Budapest, Hungary; 2 Research Centre for Natural Sciences, Institute of Cognitive Neuroscience and Psychology, Magyar tudósok körútja 2, Budapest, 1117 Hungary; 3 Integrative Neuroscience Research Group, Faculty of Information Technology & Bionics, Pazmany Peter Catholic University, Budapest, Hungary

**Keywords:** Epilepsy, Epilepsy

## Abstract

Infrared neuromodulation (INM) is a promising neuromodulation tool that utilizes pulsed or continuous-wave near-infrared (NIR) laser light to produce an elevation of the background temperature of the neural tissue. The INM-based cortical heating has been proven as an effective modality to induce changes in neuronal activities. In this paper, we investigate the effect of INM-based cortical heating on the characteristics of interictal epileptiform discharges (IEDs) induced by penicillin in anesthetized rats. Cortical heating was conducted using a NIR laser light guided through a needle-like silicon-based waveguide probe. We detected penicillin-induced cortical IEDs from preprocessed micro-electrocorticography ($\mu$ECoG) recordings, then we assessed changes in various temporal and spectral features of IEDs due to INM. Our findings show that the fast cortical heating phase obtained with continuous-wave NIR light is highly associated with a reduction of IED amplitudes, small but significant changes in the negative amplitude of IEDs compared with the baseline, and a proportional increase in the power of frequency bands related to delta/theta (2–8 Hz) and gamma (28–80 Hz) oscillations. Furthermore, a low rate of cortical heating with pulsed NIR illumination has a more inhibitory impact on the sharp negative polarity of IEDs. Our findings do not indicate a clear reduction in the frequency of IEDs in anesthetized rodents. In contrast, 2–4 min of continuous laser illumination leads to a notable increase in IED frequency. This effect of INM could potentially restrict its use in therapeutic applications related to epilepsy. However, the thermal effect of INM on cortical neurons induces changes in other characteristics of IEDs, which could prove beneficial for future applications.

## Introduction

Epilepsy is a neurological disease that can be described by sudden changes in the brain’s electrical activity that cause symptoms in brain functionality^1^. Highly synchronized electrical discharges known as interictal epileptic discharges (IEDs) appear between seizure periods^2^. IEDs occur due to the simultaneous discharge of a group of neurons in an area referred to as the epileptic focus^3^. Based on underlying neural activities, IEDs are classified into two main types^4,5^. Type-1 (IED with a slow-wave component) expresses the IEDs that correspond to the recruitment of large populations of excitatory and inhibitory cells. Type-1 is described as a spike with fast negative change and a slow positive component. Type-2 (IED without a slow-wave component) reflects the high synchrony in the underlying neuronal network and recruiting of excitatory cells in a more local area resulting in a higher negative peak amplitude. Type-2 is associated with fast ripples and results from an enhancement of synaptic excitation due to the weakening of inhibition. Moreover, type-2 IED reflects a dynamic increase in excitatory activity and the small width of this IED expresses the increased synchronization in the neural networks that are mainly supported by glutamatergic mechanisms^4^.

Neuromodulation tools and modalities such as electrical, thermal and transcranial magnetic stimulation had been developed and invested in for years to mitigate or modulate epileptic seizures and IEDs^6–8^. Thermal-based modulation of neural activity depends on the temperature-induced changes in the transmembrane capacitance and non-uniform conductance changes within the dynamics of various ion channels^9,10^. The high-resolution spatial induction of a temperature gradient using near-infrared illumination^10–14^ has been proven as an effective neuromodulation approach^15^. Infrared neuromodulation (INM) is a promising thermal-based modulation tool relying on the use of pulsed or continuous-wave near-infrared (NIR) laser light (wavelength range between 1400 and 2100 nm) to produce an elevation of the background temperature of the tissue and thus altering neuronal activities^14–16^. Xia and Nyberg illustrated that the firing rate of neurons can be reduced by 80% in a cell culture of rat cortical neurons expressing drug-induced epileptiform activities using continuous NIR light ($\lambda$ = 1550 nm, radiant exposure = 14.5 mW/mm^2^)^12^. Furthermore, during INM experiments with continuous NIR light ($\lambda$ = 830 nm, radiant exposure = 17.5 mW/mm^2^), Furuyama and colleagues reported a clear suppression in the normalized spike counts during epileptiform activity induced with bicuculline methiodide in the CA1 hippocampal region of Mongolian gerbils^13^.

The effect of hyperthermia on cultured rat cortical neurons is explained by the suppression of gamma-aminobutyric acid type B ($GABA_{B}$) receptor-mediated inhibition, which plays a significant role in hyperthermia-induced cellular hyperexcitability^17^. In addition, the underlying neural mechanism of the abnormal neuronal hyperexcitability, seizures and epilepsy belongs to the extreme effect of $GABA_{B}$ receptor-mediated inhibition^17–19^. Moreover, hyperthermia increases membrane sodium and/or calcium conductance, tissue metabolic rate and the intrinsic excitability of pyramidal cells and interneurons^20^. Accordingly, the previous findings of INM and hyperthermia give the following hypotheses. (1) Continuous wave NIR light illumination plays an influential role in suppressing epileptic discharges in terms of IED frequency^13^ and peak-to-peak amplitude of epileptiform discharges^12^. (2) In contrast, hyperthermia has an inhibitory effect on $GABA_{B}$ receptors during which the frequency of epileptiform spikes increases significantly^17^. (3) Finally, hyperthermia leads to the excitability of both excitatory and inhibitory neurons in both CA1 and CA3 pyramidal cells due to changes in the intrinsic membrane properties^20^. Based on above, this research investigates further on the thermal effect of INM by addressing the following questions:

- Can the thermal effect of NIR illumination in a drug-induced acute epilepsy model be interpreted using quantitative features extracted from epileptiform discharges?
- How much the pulsed NIR illumination at different frequencies may affect the investigated features of IEDs and thus the underlying neuronal activities?

In this study, cortical heating is carried out using an optrode probe, which is a multimodal photonic device that uses pulsed and continuous NIR light illumination to conduct a controlled cortical heating^16^. Horváth et al. have proved that this optrode device can be efficiently, repeatedly and safely used to perform deep tissue INM in anesthetized rats^16,21^. Here, infrared-based neuromodulation was performed in nine anesthetized rats. The recordings contain micro-electrocorticography ($\mu$ECoG) and temperature values of the stimulated area obtained with a pre-characterized multi-modal $\mu$ECoG array^22^. The characterization of IEDs during the INM procedure consists of loading the $\mu$ECoG datasets offline, preprocessing the signals, detecting the IEDs, extracting parametric features that represent the morphological components of IED and underlying spectral frequency bands, and then characterizing and discussing the effects of INM on the features of IEDs.

## Methods

### Animal surgery and electrophysiological recordings

All experiments were performed according to the EC Council Directive of September 22, 2010 (2010/63/EU), were in compliance with the ARRIVE guidelines, and all procedures were reviewed and approved by the Animal Care Committee of the Research Centre for Natural Sciences and by the National Food Chain Safety Office of Hungary (license number: PE/EA/672-6/2021). Acute in vivo experiments were carried out on Wistar rats (n = 9; weight: 300.56 g ± 65.79 g, mean ± standard deviation; 6 females). A mixture of intramuscularly administered ketamine (75 mg/kg) and xylazine (10 mg/kg) was used to induce anesthesia. During the electrophysiological recordings, supplementary doses of ketamine/xylazine were injected intramuscularly to maintain the anesthesia. A homeothermic heating pad connected to a temperature controller (Supertech, Pécs, Hungary) was used to keep the physiological body temperature of the animals. After reaching the appropriate anesthetic depth for surgery, the head of the rat was fixed in a stereotaxic frame (David Kopf Instruments, Tujunga, CA, USA), then the skin and the connective tissue was removed from the top of the skull. Next, a cranial window with a size of about 10 mm $\times$ 5 mm was drilled over the left brain hemisphere (anterior-posterior [AP]: from +1 mm to $-9$ mm; medial-lateral [ML]: from 0.5 to 5.5 mm; coordinates given with respect to the bregma^23^). The dura mater was left intact during the whole experiment except in a few cases (n = 3) where the bone could not be removed from the craniotomy without tearing the dura, as they were tightly fused.

**Figure 1:**
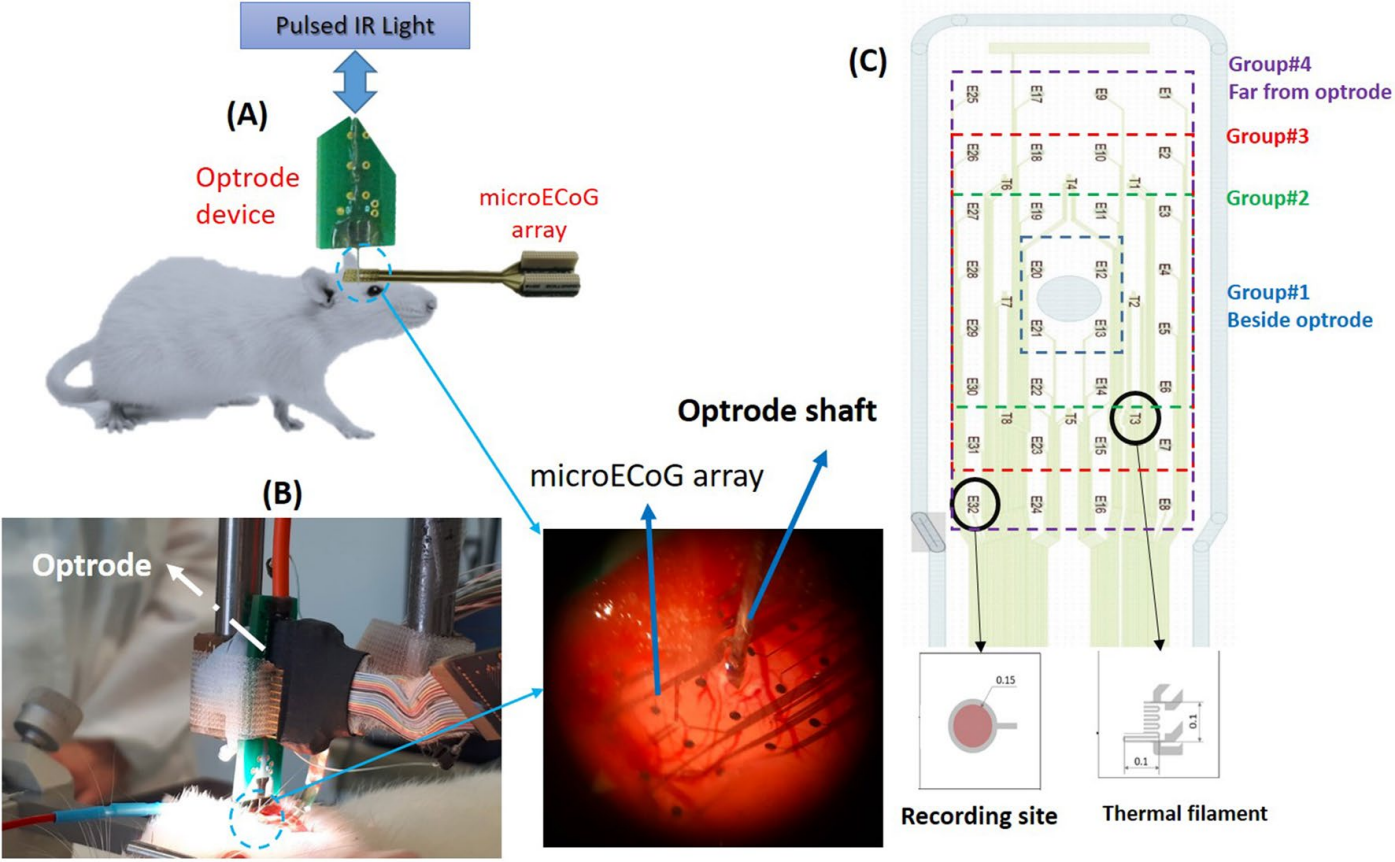
In vivo neuromodulation experiment and tools. (**A**) Experimental setup^22,24^; (**B**) In vivo neurosurgery; (**C**) Layout of the $\mu$ECoG array with 32 electrophysiological recording sites (E1-E32) and 8 temperature filaments (T1–T8) in addition to labelled and grouped recording sites.

A flexible, polyimide-based $\mu$ECoG array^22^ with 32 platinum recording sites (150 $\upmu$m diameter) and 8 miniature thermometers (100 $\upmu$m $\times$ 100 $\upmu$m) was mounted on a micromanipulator, then carefully lowered into the cranial window and placed on the exposed cortical surface (Fig. 1A). In its middle, the $\mu$ECoG array has a circular aperture with a diameter of 800 $\upmu$m dedicated for the insertion of implantable devices. Small pieces of hemostatic sponge were carefully placed on the top of the array around the aperture. After that, room temperature physiological saline solution was dripped on the sponge to prevent dehydration of the cortical tissue as well as to provide mechanical stabilization for the $\mu$ECoG array. A silicon-based optical probe (optrode;^16,21,24^) designed for infrared stimulation (see “Infrared stimulation protocol” section for detailed information) was inserted through the aperture (Fig. 1B) into the brain tissue to a depth of 1.2 mm with a slow speed (2 $\upmu$m/s; to decrease the insertion-related mechanical tissue damage^25^). A stainless-steel needle inserted in the nuchal muscle of the animal served as the reference and ground electrode during recordings.

Cortical electrical activity was collected using an Intan RHD2000 electrophysiological recording system (Intan Technologies, Los Angeles, CA, USA) equipped with a 32-channel headstage (for the $\mu$ECoG recordings). Wideband signals (0.1–7500 Hz) were acquired with 20 kHz/channel sampling rate and with 16-bit resolution. Electrophysiological data were saved to a local network attached storage device for offline analysis. The temperature of the cortex was recorded using the thermal filaments of the $\mu$ECoG array with a separate device based on an Arduino Mega2560 with a sampling frequency of 6 Hz. To avoid any additional noise that may contaminate the recording of $\mu$ECoG signals, the temperature recordings of the cortex has been conducted at the end of the experiment after finishing the $\mu$ECoG recording, using the same INM protocol parameters.

### Infrared stimulation protocol

The optrode used in the experiments is a multimodal photonic neural probe that provides high spatial and temporal control of temperature elevation using an embedded infrared waveguide^21^. The fabrication of this device was presented in detail in^24^, and results of the in vivo testing of the device were published in^16,21^. The device has a 5 mm long, 170 $\upmu$m wide and 190 $\upmu$m thick implantable shaft which acts as an optical waveguide. The silicon waveguide emits the NIR light at the needle-like sharp tip of the shaft. The INM process consists of delivering NIR light from a pig-tailed NIR laser diode (LPSC-1550-FG105LCA-SMA, Thorlabs, Inc., USA) with a nominal optical power of 70 mW and a wavelength of 1550 nm through the optrode shaft. In this research, we applied two, slightly different NIR stimulation protocols (ISPs). The first protocol (ISP1) composes of 2-min of control (baseline of early starting, labeled PCtrl), 2-min of NIR stimulation (diode switched on, labeled P2ON), and 4 min without NIR stimulation (diode switched off, labeled P4OFF). The second protocol (ISP2) is similar to ISP1 but with 4 min of NIR stimulation (P4ON). In a single animal, the ISP was repeated several times, each time using a different pulse frequency. For each pulse frequency, we first recorded baseline activity for 2 min (PCtrl) then carried out 5 repeated trials of INM (P2ON/P4ON then P4OFF, 5 times after each other). Driving current of the IR laser diode was supplied by a Keithley 2611B source measure unit (Keithley Instruments Inc, OH, USA). The current output of the source measure unit was switched on and off with a duty cycle of 50% and with the following pulse frequencies: 0 Hz (continuous); 1 Hz; 10 Hz; 100 Hz; 1 kHz.

### Induction of epileptiform activity

To induce IEDs, penicillin-G (P3032, Sigma Aldrich, Saint Louis, MO, USA) was injected into the brain using a 34-gauge needle. The needle attached to a Hamilton syringe was mounted on a micromanipulator, then slowly (20 $\upmu$m/s) lowered to a cortical depth of 1.5 mm through the aperture of the $\mu$ECoG array at an angle of 30$^\circ$ from vertical. Next, 1 $\upmu$l of penicillin (300 IU/$\upmu$l) was injected into the cortex at a rate of 0.2 $\upmu$l/min. Epileptiform spikes appeared a few minutes after injection. After that, the injection needle was retracted and the infrared stimulation protocol was started along with the electrophysiological recordings.

### Signal preprocessing

$\mu$ECoG signals were preprocessed offline using Matlab (MathWorks, Natick, MA, United States). A second-order notch filter (50 Hz) was used to remove power line noise, and a second-order Butterworth band-pass filter (2–300 Hz) was used to keep only frequencies related to IEDs. A second-order Butterworth band-stop filter (80–120 Hz) was used to filter out the artefacts caused by the NIR stimulation equipment. The recording sites of the $\mu$ECoG array were labeled based on the distance from NIR illumination (optrode aperture) into four main groups, where “Group#1” refers to the four recording sites around the optrode and “Group#4” refers to the furthest sites from optrode (Fig. 1C). Bad channels (n = 16 from the 32) were identified by visual inspection of the recordings and not used for further analysis.

### IED detection

Interictal discharges were detected using a custom Matlab script. Our IED detection approach had the following steps: (1) filtering the recording site’s signal in the 30–300 Hz range using a second-order Butterworth band-pass filter because this frequency range represents the most dominant band that contains the pure interictal discharges^26^; (2) using a 10-second-long sliding window with 0.25-sec of overlap between windows, we calculate the square signal of the window to differentiate between IED and background activity easier, then we compute the local-maxima peaks within the squared signal; (3) an adaptive threshold is computed which equals the average of detected local-maxima peaks within the squared signal and the average of the squared signal set; (4) the adaptive threshold is used to detect IEDs. In summary, the IED detection method depends on calculating the detection threshold adaptively in a sliding 10-sec window. This method showed an accuracy of 95$\sim$100% by detecting IEDs manually in 100 randomly selected 10-second-long windows and calculating the error in detected IED count using manual detection and the proposed automatic detection method.

### IED feature extraction

The feature extraction stage is implemented directly for each detected IED by isolating the IED from the filtered (2–300 Hz) signal using a 300 ms-long segment. The center of the thresholded part of square signal (step $\#$2 in IED detection stage) represents the center of the detected IED. The detected IED was extracted using ±150 msec frame from its center. The IED features involve (1) peak-to-peak amplitude of IEDs^27,28^ that expresses the synchrony state of the underlying neuronal network^4^; (2) the ratio of the negative-peak (or local minima) to the peak-to-peak amplitude^27,28^ which is positively correlated to the recruitment rate of excitatory cells^4,5^; (3) the ratio of delta and theta (2–8 Hz) band power to the (2–300 Hz) band power^29,30^ obtained using power spectral density (PSD), where increased delta and theta synchronization refers to cortical dysfunction thus the decreased ratio can be interpreted as partial normalization of cortical activity^31,32^; (4) the ratio of gamma (28–80 Hz) band power to the 2–300 Hz band power using PSD, where increased gamma results from the dysfunctional state of the underlying multiple ion channels or neurotransmitter receptors in awake subjects^31^; (5) IED frequency that is calculated from the total count of detected IEDs for every 40 seconds. In this study, the clustered IED frequency during each 40-sec time-frame was sufficient for expressing the IED occurrence of each INM phase (e.g. first 40 seconds expresses the heating-up phase, see “Temperature of the cortex during INM” section in Results for more details) with less visual data.

### Statistical analysis

The values of each extracted feature per trial/rat/frequency/site group are formulated as change rate per 40 seconds during INM phases (PCtrl, P2ON/P4ON and P4OFF), then normalized to the change rate of the last 40 seconds of PCtrl in array form called X with (ISP1: 12; ISP2: 15 elements) length. To measure the similarity of each X array among trials/rats, Pearson correlation was implemented between the average of all X arrays (trials and rats using the same frequency and same site group) and the average of X arrays (only trials using the same frequency and same site group of the same rat). The final correlation ($\rho$) represents the average of all pairwise X combinations where the negative correlation was considered as zero value, ($0 \le \rho < 0.4$) was considered a weak positive correlation, ($0.4 \le \rho < 0.8$) as moderate and ($0.8 \le \rho \le 1.0$) as strong positive correlation. By making the negative correlation equal zero^33^ before calculating the average correlation of pairwise X combinations, we make sure that any reverse change of feature values among trials will decrease the significance of our results. The strong positive correlation of ($\rho$) value expresses the high similarity among trials/rats with the same circumstances and parameters. The statistical significance of the correlation (*p* value) of each pairwise X combination expresses the reliability of that correlation and its importance. All statistical processes were computed using descriptive statistics functions in MATLAB. The null hypothesis shows that there is no relationship and there is no similar change of feature during laser ON and OFF phases among rats and trials with the same protocol. Changes with *p* value < 0.05 are considered statistically significant.

## Results

### Temperature of the cortex during INM

**Figure 2:**
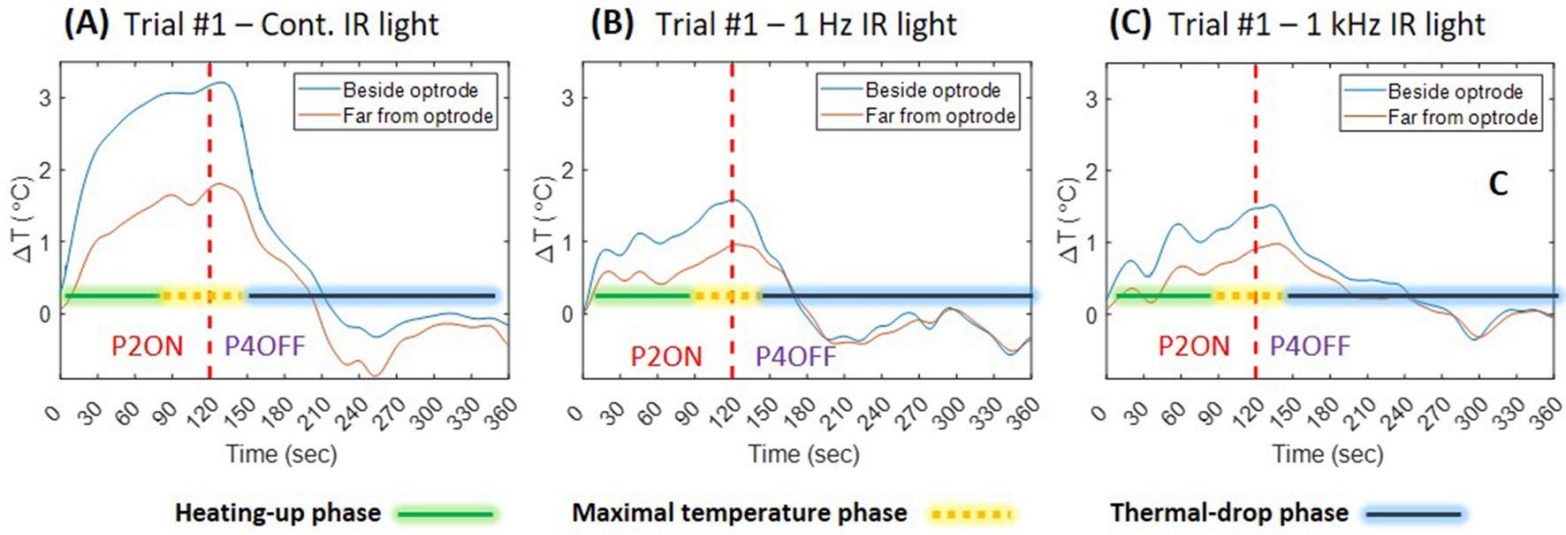
The main phases of cortical temperature changes measured at two positions (close to and far from optrode) during ISP1 protocol with (**A**) continuous-wave; (**B**) 1 Hz-pulsed; (**C**) 1 kHz-pulsed NIR light. The blue curve refers to the temperature of the cortex beside optrode, the red curve refers to temperature changes of the cortex using the thermal filament furthest from optrode and the red dashed-line separates P2ON (infrared stimulation ON) and P4OFF (infrared stimulation OFF) phases.

The INM protocol contains five sessions, and each session is related to a specific illumination frequency. The used $\mu$ECoG array contains eight thermal filaments, two of these filaments are located close to the penetrating optrode (T2 and T7, Fig. 1C) and the rest are distributed symmetrically in two rows around the $\mu$ECoG array aperture. In this study, we used the temperature recorded by two filaments (close and far from the optrode). The thermal filament (T2) located close to the optrode detected the highest temperatures, while the filament (T8) further away from the optrode detects lower changes. The temperature changes of the cortex during each phase of the INM protocol at a specific frequency of NIR light are illustrated in Fig. 2. The continuous-wave NIR light achieved the highest temperature change of ($\sim$3 $^\circ {\hbox {C}}$) close and ($\sim$1.5 $^\circ {\hbox {C}}$) far from the optrode compared with other frequencies (Fig. 2A; P2ON). Also, there is a stable decrease of temperature during laser OFF periods (P4OFF). The temperature of the cortex showed the smallest changes during 1 Hz pulsed NIR light with a maximum increase of about 1.6 $^\circ {\hbox {C}}$ close to and 0.8 $^\circ {\hbox {C}}$ far from the optrode, respectively (Fig. 2B), and fluctuations of cortical temperature during P4OFF phase. We hypothesize that the latter is related to the co-occurring cooling effect of the convection of the ambient air and the effect of hemodynamics that may have more influence at lower cortical temperatures. During 1 kHz pulsed NIR light, the temperature of the cortex tends to show a stable increase and decrease with a similar time course like measured during the continuous NIR illumination but with lower temperature changes that equal to $\sim$1.5 $^\circ {\hbox {C}}$ (Fig. 2C). The distinguishing changes in temperature during INM protocols (concerning laser ON and OFF phases) can be categorized into three phases (Fig. 2 shows the labelled phases of cortical temperature during ISP1 protocol): (1) The heating-up phase describes the elevating temperature of the cortex during laser ON. (2) The steady maximal cortical temperature phase describes the short period of stable and maximal cortical temperature until the temperature starts dropping shortly after NIR stimulation has stopped (P4OFF). (3) The thermal-drop phase of the cortex and returning to baseline temperature.

### Changes in IEDs during INM

**Figure 3:**
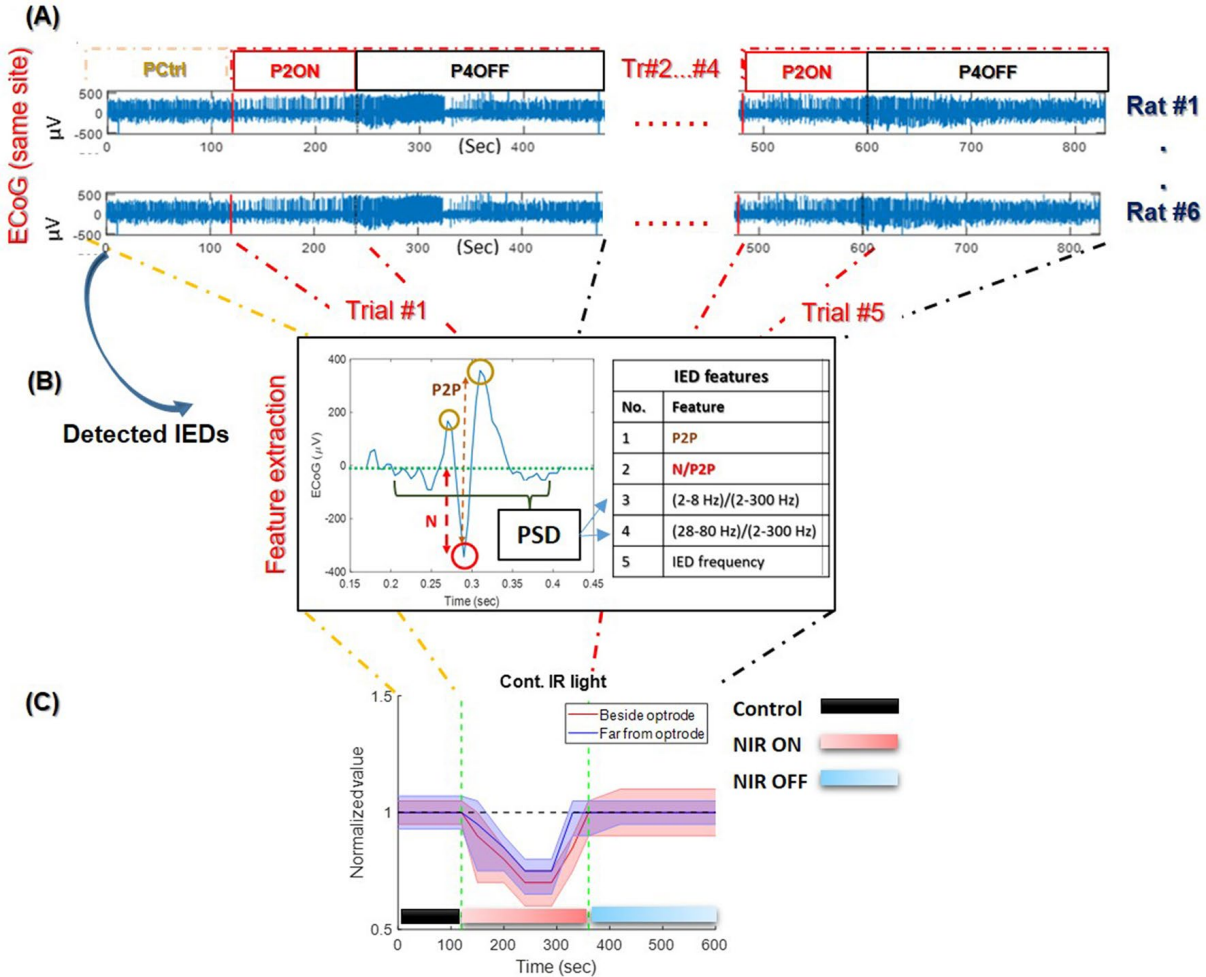
Calculation and representation of extracted features of detected IEDs from $\mu$ECoG signals during the same frequency of NIR light. (**A**) divide the same $\mu$ECoG signals of all rats and trials into PCtrl and 5 trials of (laser ON and OFF) phases; (**B**) feature extraction stage of detected IED; (**C**) representing each extracted feature as an average and a standard deviation of all rats and trials in 3 phases of INM with the same protocol where the horizontal axis represents the time and the vertical axis represents the normalized value of the feature.

**Figure 4:**
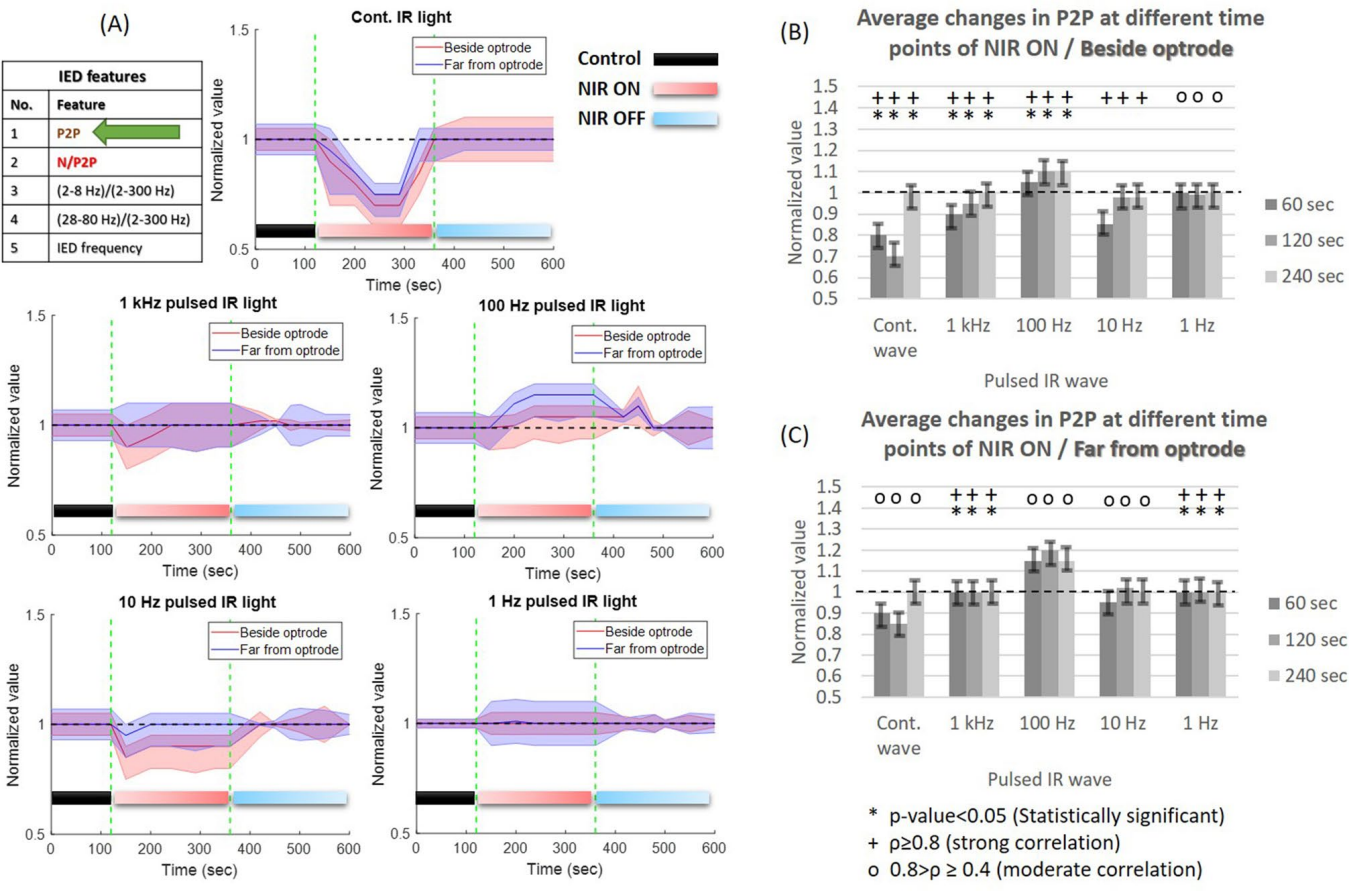
Normalized changes in the peak-to-peak (P2P) amplitude of IEDs in anesthetized rats (ISP1 and ISP2 protocols were performed on six and three rats, respectively) during different frequencies of NIR light at two recording sites of the $\mu$ECoG array. (**A**) Dashed green lines separate between PCtrl, laser ON and OFF phases respectively, solid lines represent averages, shaded regions indicate standard deviations (SDs); (**B**) average changes of P2P amplitudes of IEDs beside optrode at different time points of NIR stimulation. Whiskers represent SDs; (**C**) average changes of P2P amplitudes of IEDs far from optrode.

**Figure 5:**
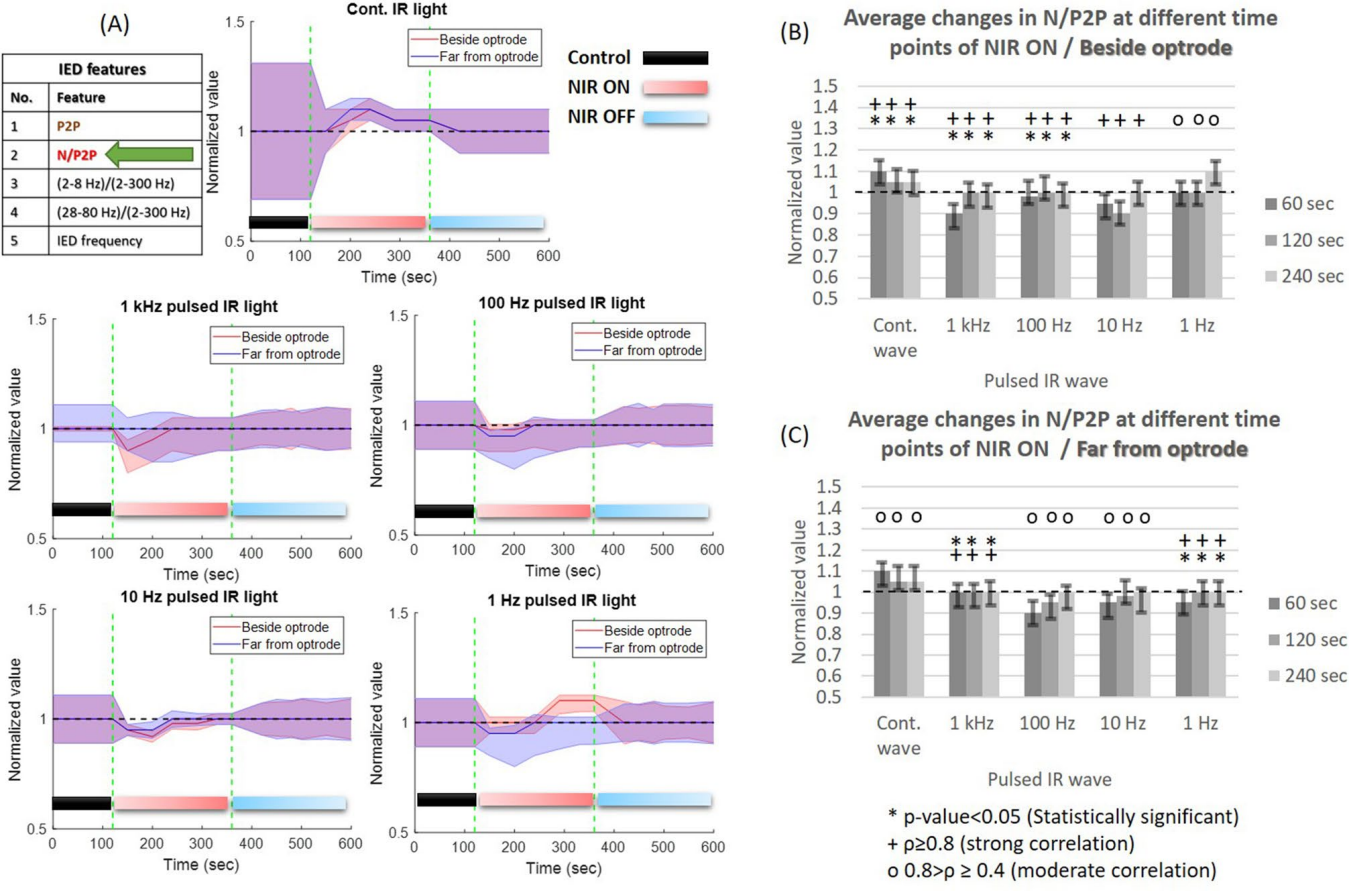
Normalized changes in the negative (N/P2P) amplitude of IEDs in anesthetized rats (cohort size is the same as in Fig. 4) during different frequencies of NIR light at two recording sites of the $\mu$ECoG array. The features of the figure are the same as in Fig. 4.

**Figure 6:**
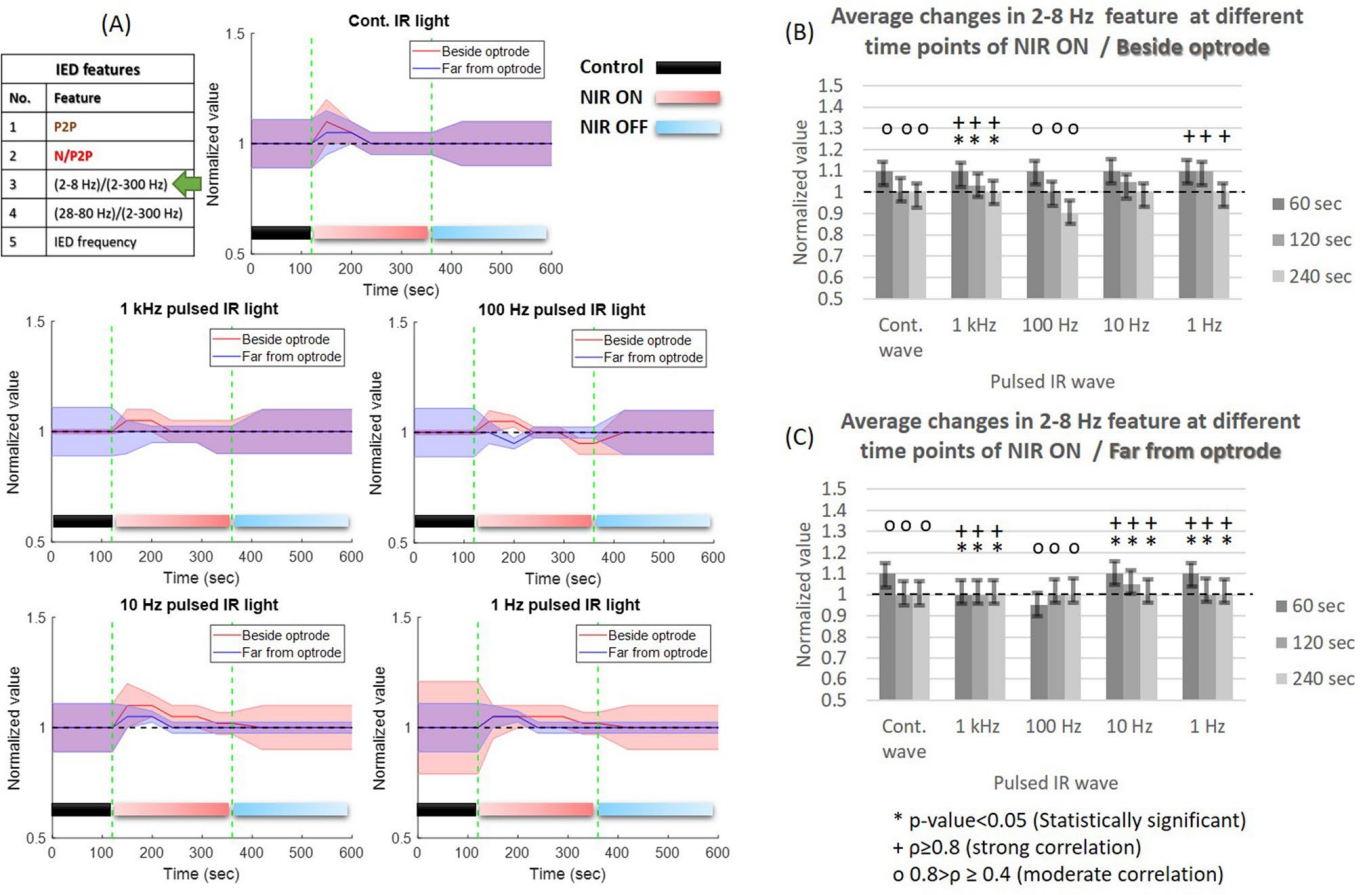
Normalized changes in the ratio of (2–8 Hz) band power to the total band (2–300 Hz) power of IEDs in anesthetized rats (cohort size is the same as in Fig. 4) during different frequencies of NIR light at two recording sites of the $\mu$ECoG array. The features of the figure are the same as in Fig. 4.

**Figure 7:**
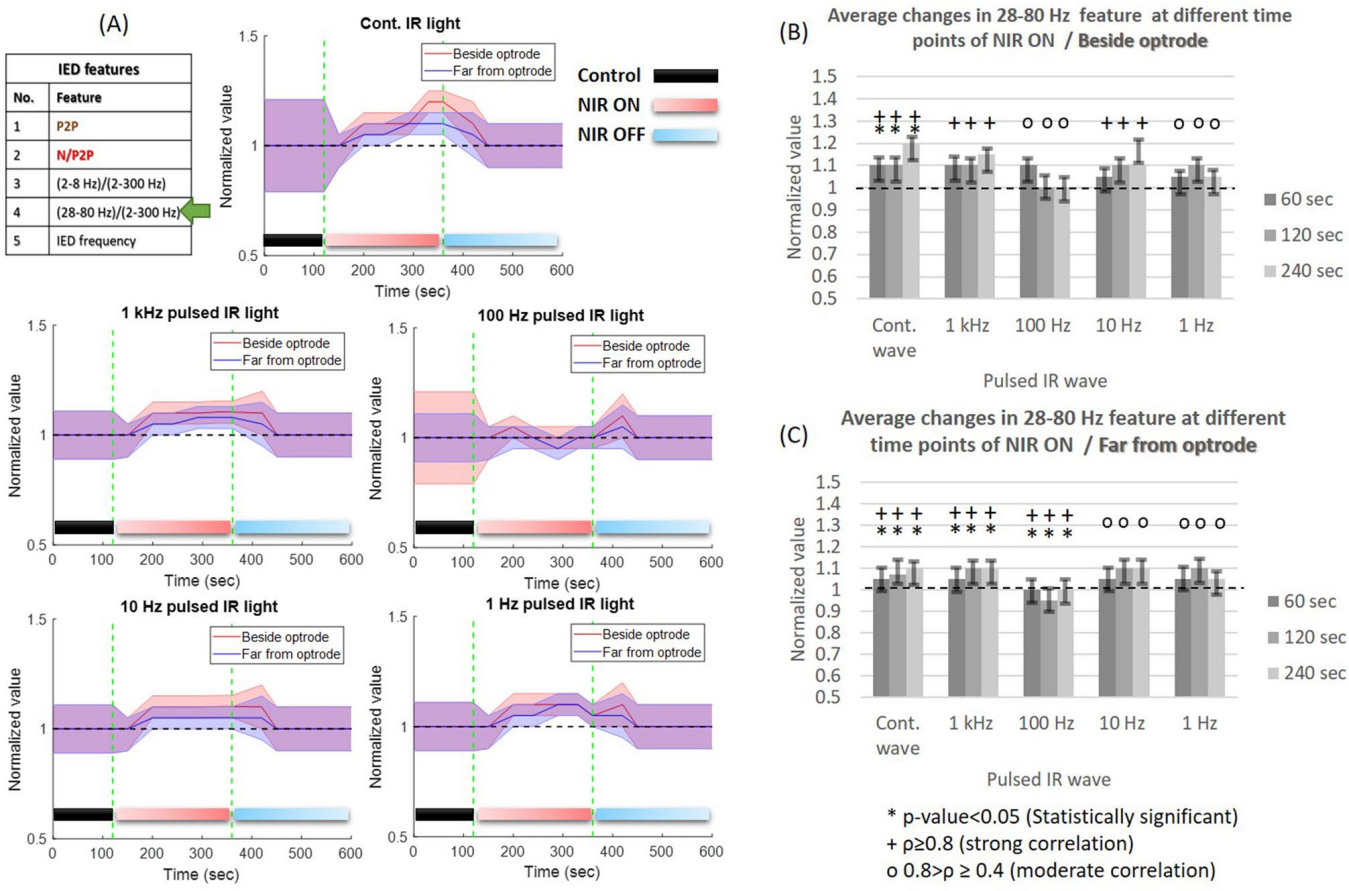
Normalized changes in the ratio of (28–80 Hz) band power to the total band (2–300 Hz) power of IEDs in anesthetized rats (cohort size is the same as in Fig. 4) during different frequencies of NIR light at two recording sites of the $\mu$ECoG array. The features of the figure are the same as in Fig. 4.

**Figure 8:**
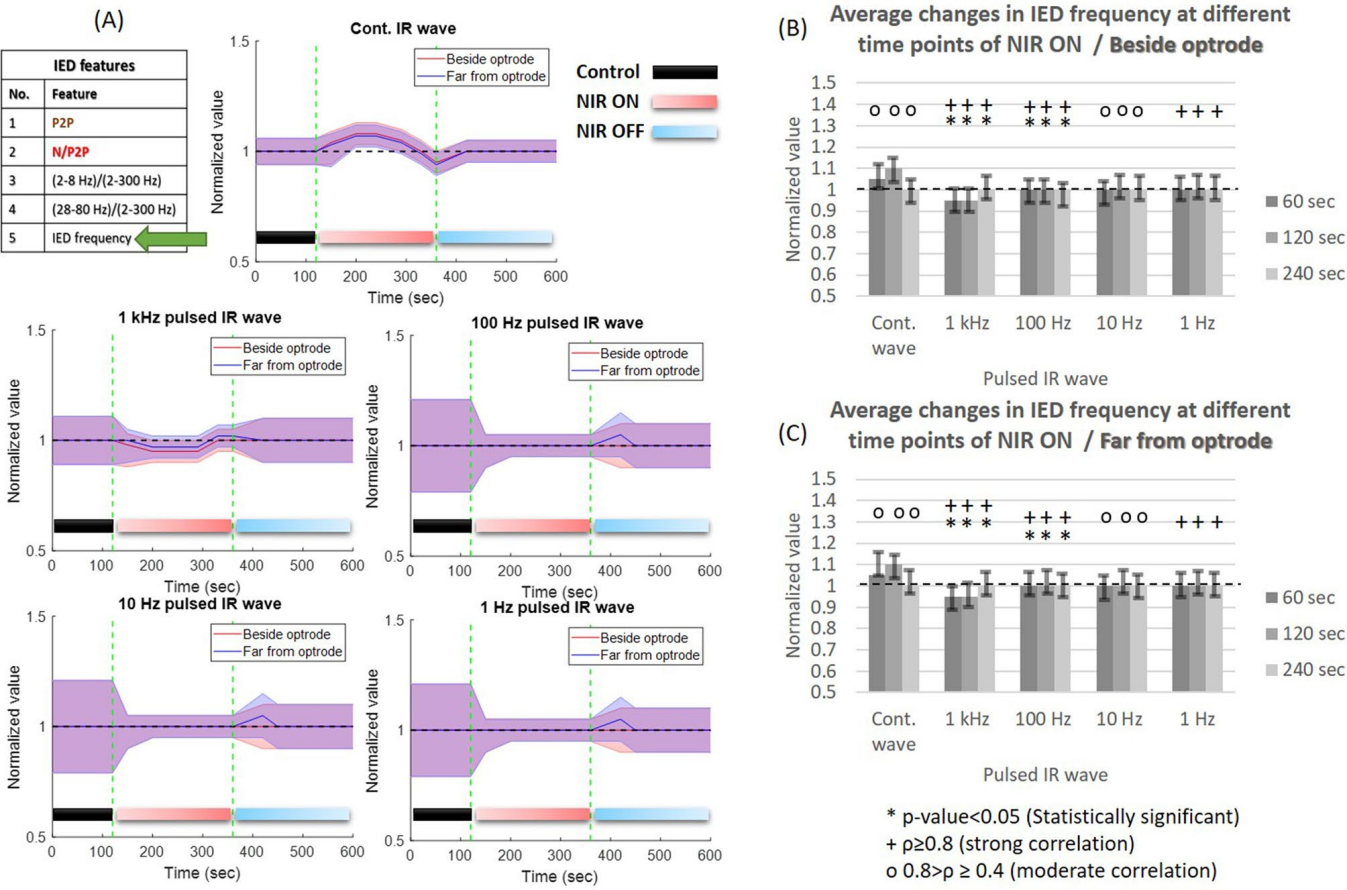
Normalized changes in IED frequency in anesthetized rats (cohort size is the same as in Fig. 4) using different frequencies of NIR light at two recording sites of the $\mu$ECoG array. The features of the figure are the same as in Fig. 4.

Our INM protocols performed on nine anesthetized rats involved the use of continuous, 1 Hz, 10 Hz, 100 Hz and 1 kHz pulsed NIR light. The five configurations of pulsed NIR light with ISP1 and ISP2 protocols were performed on six and three rats, respectively. All detected IEDs were characterized using five extracted features (see the “Methods” section for details) that reflect the changes in neuronal activities during INM experiments. The proposed approach of analyzing data and visualizing the extracted features is demonstrated in Fig. 3, including $\mu$ECoG signals and IED detection (Fig. 3A), IED feature extraction (Fig. 3B) and visualizing the changes as average and standard deviation among rats and trials (Fig. 3C). In this section, two recording site groups (Group#1 and Group#4) of $\mu$ECoG during ISP1 and ISP2 protocols are considered for all frequencies of NIR light (Figs. 4, 5, 6, 7, and 8). Detailed graphs of the five features and four site groups of both ISP1 (Figs. A1.2–A1.6) and ISP2 (Figs. A2.2–A2.6) protocols are provided in the supplementary documents.

#### Changes in peak-to-peak amplitude of IEDs

*Frequency-related changes:* the normalized change of peak-to-peak amplitude during continuous wave NIR illumination (Fig. 4A) shows reduction during stimulation in all $\mu$ECoG sites especially beside optrode ($\rho =$ 0.87, *p* value = 0.03) about 20-30% amplitude suppression compared to the baseline but the reduction is smaller at the furthest recording sites ($\rho =$ 0.75, *p* value = 0.16) about 0–15% of suppression (Fig. 4B,C). From 1 kHz to 1 Hz of pulsed NIR light, the heating-up phase has less influence on the amplitude of the IED. The changes of peak-to-peak amplitude beside optrode (Fig. 4B) show a strong positive correlation along trials ($\rho >80\%$; *p* < 5$\%$). Recording sites far from the stimulus location (Fig. 4C) mostly show moderate correlation along trials so there is no sufficient similarity among trials.

*Heating phase-related changes:* the changes in peak-to-peak amplitude can be alleviated only with a high temperature and fast heating-up phase. The slow rate of cortical heating with 1–100 Hz pulsed NIR illumination leads to a fluctuation in peak-to-peak amplitude and a mild increase in IED amplitude compared with PCtrl. Keeping the steady state of maximal temperature for a longer period of time, we can maintain heating-up impact on IED amplitude (Fig. 4). The thermal-drop phase during INM trials and frequencies always leads to gradual return the baseline with some delay after the heating is turned off.

#### Changes in the negative peak of IEDs

*Frequency-related changes:* the normalized change of negative peak with continuous-wave NIR light (Fig. 5) shows a proportional increase during laser ON phase (beside optrode: $\rho =$ 0.87, *p* value = 0.03; far from optrode: $\rho =$ 0.75, *p* value = 0.16) but that increase does not exceed the values of PCtrl phase. Using 1 kHz pulsed NIR light, negative-peak feature tends to slightly decrease (Beside optrode: $\rho =$ 0.98, *p* value = 0.01; Far from optrode: $\rho =$ 0.91, *p* value = 0.01) during laser ON phase. During 1 Hz pulsed NIR light, negative-peak feature shows a constant rate of change beside optrode ($\rho =$ 0.86, *p* value = 0.08) and a clear decrease in the furthest channel (Fig. 5B,C). The changes of negative peak show a strong positive correlation beside NIR light illumination as shown in (Fig. 5B). In contrast, those changes show more fluctuations and instability with moderate correlation in the furthest sites (Fig. 5C).

*Heating phase-related changes:* compared to the baseline and laser OFF phase, the cortical heating maintains the fluctuation of negative amplitude and sometimes mitigates it. Commonly, there are narrow fluctuations rather than notable reductions in the negative-peak feature during INM protocols compared to the PCtrl. In addition, the steady maximal cortical temperature can maintain the stability of N/P2P ratio.

#### Changes in the 2–8 Hz band power of IEDs

*Frequency-related changes:* the 2–8 Hz band-power feature expresses the ratio of 2–8 Hz to 2–300 Hz band power of detected IEDs. The normalized change of this feature with continuous-wave NIR light (Fig. 6) shows increase in 2–8 Hz component during laser ON in all channels without statistical significance ($\rho =$ [0.71, 0.76], *p* value = [0.18, 0.2]). With 1 kHz pulsed NIR light, the effect on the 2–8 Hz frequency band becomes weaker (beside optrode: $\rho =$ 0.92, *p* value = 0.01; far from optrode: $\rho =$ 0.93, *p* value = 0.01), and with 1 Hz of NIR light there are narrow changes (more stable) in 2–8 Hz band power ($\rho =$ 0.84, *p* value = 0.1) and this effect becomes dominant with 4 mins NIR ON. Beside NIR laser illumination, there is a moderate positive correlation (Fig. 6B), while in the furthest recording sites, the changes of 2–8 Hz band-power show a strong positive correlation among trials (Fig. 6C).

*Heating phase-related changes:* the power of delta and theta bands of IED increases proportionally during heating-up phase while tending to decrease and stable in a narrow range of changes during the maximal steady cortical temperature with moderate statistical significance as shown in (Fig. 6A). The long-term maximal temperature also proves the stability of reduction for 100-1000 Hz and increase for 1–10 Hz in 2–8 Hz band power (Fig. 6).

#### Changes in the 28–80 Hz band power of IEDs

*Frequency-related changes:* the normalized change of 28–80 Hz band power with continuous-wave NIR light (Fig. 7) illustrates the proportional increase during laser ON (beside optrode: $\rho =$ 0.91, *p* value = 0.01; far from optrode: $\rho =$ 0.95, *p* value = 0.01). This elevated state of gamma activity is maintained at 1 kHz pulsed NIR light (Fig. 7B,C) beside optrode’s region (beside optrode: $\rho =$ 0.85, *p* value = 0.07) and far from optrode (far from optrode: $\rho =$ 0.9, *p* value = 0.02). At pulsed illumination, a moderate increase in bandpower can be observed during the stimulus onset, which is more dominant as the stimulus onset time is increased. *Heating phase-related changes:* the induction of gamma oscillation is obvious during heating up phase and this elevated state is maintained at the maximal steady cortical temperature (Fig. 7B,C).

#### Changes in IED frequency during INM

*Frequency-related changes:* the normalized change of IED frequency with continuous wave NIR light (Fig. 8) shows a proportional increase in IED frequency during NIR illumination phase of ISP1/ISP2 (beside optrode: $\rho =$ 0.68, *p* value = 0.17; far from optrode: $\rho =$ 0.68, *p* value = 0.17) where the correlation and *p* value show instability in IED frequency among rats and trials so there is no clear evidence about the dominant change in IED frequency. With 1 kHz pulsed NIR light (Fig. 8B,C), the IED frequency during NIR ON decreases compared to PCtrl and P4OFF phases (beside optrode: $\rho =$ 0.90, *p* value = 0.02; far from optrode: $\rho =$ 0.9, *p* value = 0.02) and with 1–100 Hz frequency of pulsed NIR light there is no clear evidence about the dominant change in IED frequency. At 1–100 Hz illumination, in addition to the long-term maximal temperature (Fig. 8A), there is neither inhibitory nor excitatory effect but the changes of IED frequency became more tight. Regarding the IED frequency, there are no clear inhibitory or excitatory effects of INM on this feature.

## Discussion

The mechanism of thermal-based neuromodulation is based on inducing a temperature gradient (dT/dz or dT/dt) or boosting the temperature of the tissue^15^. This induction elicits or suppresses the action potential of neurons^14,16,34,35^ that results from changes in the transmembrane capacitance and non-uniform changes in the conductance dynamics of specific ionic channels^36^. The modulation of both wavelength and the power of continuous-wave NIR illumination leads to efficient suppression of induced epileptic seizures^12^. Moreover, INM was able to mitigate spontaneous neural activity^35^ and induce excitatory activities in superficial cortical neurons^37^. In this research, we investigated the effect of cortical heating on the properties of IEDs in anesthetized rats. The cortical heating was carried out using a needle-like sharp tip silicon waveguide probe that transmits continuous-wave and pulsed-wave into the tissue and measures the evoked changes in the epileptiform activity through integrated recordings sites^21^. Various stimulation protocols comprising 2-mins ON and 4-mins OFF periods using the same optrode were conducted on anesthetized rodents and have caused a permanent increase in the firing rate of cortical neurons in control subjects^16^. Moreover, Xia et al.^12^ used 30-sec continuous-wave NIR to modulate the neuronal activities in rat cell cultures. Based on these findings, we proposed that 2–4 min of pulsed NIR illumination is sufficient to examine the effects of the IR wave during the designated period without causing any damage to the tissue^21^.

*Frequency-related changes:* the normalized changes of the peak-to-peak amplitude of IEDs show an apparent reduction in the amplitude ($\sim$25%) when continuous NIR stimulation was used (Fig. 4). This reduction becomes less pronounced with low-frequency pulsed NIR light (Fig. 4). The decrease in peak-to-peak amplitude during continuous-wave NIR illumination agrees with the previous findings of inhibiting epileptic seizures^12^ and neural activity^34,35^. On the other hand, the low-frequency pulsed-IR waves induce lower temperatures and a slow rate of heating up that leads to no change or mild-increase of IED amplitude. The mild excitatory effect of low-frequency pulsed NIR waves corresponds to the finding about the contribution of moderate heat in synchronizing brain networks using INS procedure in the visual cortex ($\lambda$ = 1.875 $\upmu$m, pulse width = 250 $\upmu$s, radiant exposure = 0.57 J/cm^2^, repetition rate = 200 Hz, stimulation duration = 500 ms)^37^. The mild increase in peak-to-peak amplitude (Fig. 4) and stability in negative amplitude (Fig. 5) during cortical heating is considered evidence of induced slow component in IED. Accordingly, the slow rate of cortical heating and low values of maximal temperature using 1–1000 Hz pulsed NIR light reduce the recruitment rate of excitatory cells^4,5^ due to their association with inducing the slow component.

*Heating phase-related changes:* the amplitude suppression of IEDs during INM is associated with the high and fast rate of cortical heating which only occurred using continuous-wave and 1 kHz NIR illumination. Our study emphasizes the inhibitory effect of continuous, 1 kHz and 10 Hz NIR illumination on IED amplitude, especially during the heating-up phase. In addition, the steady maximal cortical temperature phase leads to a mild increase in IED amplitude. The normalized change of the negative-peak feature between the PCtrl and INM phases shows narrow fluctuations during the heating-up phase using continuous wave NIR (Fig. 5) which refers to stability in the synchrony of the cortex^4^. The normalized change of 2–8 Hz band power to the 2–300 Hz band power shows a proportional increase during P2ON and P4ON of the protocols (Fig. 6) especially during heating-up phase. During the steady maximal temperature of INM protocols, the power of delta and theta bands decreases proportionally. Both the low rate of cortical heating and thermal drop cause a normalization of cortical activity^31,32^. Gamma (28–80 Hz) band power clearly increases during the P2ON and P4ON phases, where both INM protocols produce an induction in gamma rhythm (Fig. 7). This elevated state gamma activity is maintained at steady maximal cortical temperature phase. The most important finding is that the increase in gamma band power is fully associated with the reduction in peak-to-peak amplitude and the narrow fluctuations of the negative amplitude of IED (Figs. 4, 5 and 7). The normalized IED frequency feature (Fig. 8) during the cortical heating phase of continuous wave NIR illumination induces more IEDs. The heating-up phase using 1–1000 Hz pulsed laser illumination contributes more to inhibiting or mitigating the IED occurrence. The steady maximal temperature during trials does not illustrate any consistent change in IED frequency. Previous findings reported that the spike rate is suppressed effectively^12,13^ but the lowest power of continuous-IR illumination did not show a notable inhibiting effect^12^. The increase in IED frequency apparently agrees with previous work^20,37^ about the role of hyperthermia in increasing the IED frequency. This inconsistency in the effect of INM on IED frequency can be explained as the dual role of cortical heating in inhibiting the local cortex activity^12,16,35^ and repression of $GABA_{B}$ receptor-mediated inhibition responsible for cellular hyperexcitability and epileptiform discharges^17–19^. Some of the changes, such as the reduction in 2–8 Hz band power and negative amplitude of IEDs, provide evidence that prolonged NIR stimulation can lead to notable changes in neural activities. Since optrodes have been designed and validated^16,21^ for extended periods of NIR stimulation and $\mu$ECoG arrays have been applied for long-term experiments^22^, it is worth to focus on examining the impact of thermal stimulation using the optrode with varying durations of IR illumination.

Our findings do not indicate a clear reduction in the frequency of IEDs in anesthetized rodents. In contrast, 2–4 min of continuous laser illumination leads to a notable increase in IED frequency. This effect of INM could potentially restrict its use in therapeutic applications related to epilepsy. Previously, studies reported a decrease in IED frequency through the use of continuous-wave NIR stimulation. These studies were conducted on cell cultures of rat cortical neurons^12^ and in the CA1 hippocampal region of Mongolian gerbils^13^. Our findings show that the thermal effect of NIR stimulation has different effects on the cortex of anesthetized rodents with penicillin-induced IEDs. Moreover, our study investigates the long-period thermal effect of NIR stimulation on changes in various temporal and spectral features of IEDs. The NIR stimulation was able to modulate various characteristics of IEDs, including an increase in 2–8 Hz and 28–80 Hz band power of IEDs. These findings hold promising potential for prospective applications in the future^31,32^. Concerning the generalizability of the results, the low standard deviations of normalized values in Figs. 4, 5, 6, 7, and 8 express the generalizability of the results in exact time points. To confirm this point, the results must be enriched with other experiments so that the randomness of the samples is taken into account.

Pulsed NIR light modulation may provide prospective therapeutic approaches after a deeper investigation of its effects in freely moving rodents. The penetrating optrode tool used in this study had been extensively tested previously in acute experiments in anesthetized rats^16^ and validated using in vitro characterization tools^24^. In addition, this type of optrode did not cause any significant histological damage to the cortex of rodents^21^. By incorporating additional accessories, this optrode can be securely fixed and chronically inserted into the cortex of freely moving rats, allowing to assess the long-term effects of infrared stimulation. $\mu$ECoG arrays had also been validated for chronic cortical recordings in awake, behaving rodents using long-term in vitro and in vivo characterization^22^. The collected data can be subsequently processed and analyzed offline using the required hardware and software.

## Conclusion

This paper investigates the INM-based cortical heating procedure and its effect on interictal discharges in a penicillin-induced acute model. With the use of the multimodal photonic device we successfully achieved precisely controlled cortical heating experiments using a laser diode source. By characterizing the detected IEDs in $\mu$ECoG signals during INM experiments using the extracted features, we were able to analyze and express the morphological changes of IEDs and the power of underlying spectrum bands. In summary, the investigated thermal effects of 2–4 min of pulsed NIR laser illumination on IEDs in anesthetized rodents are as follows:

- The fast heating-up phase of continuous-wave INM is highly associated with mitigating the amplitude of IEDs. On the other hand, using 1 kHz, 100 Hz and 1 Hz of pulsed NIR light, the heating-up phase has less influence on the amplitude of the IEDs, where there was no observable decrease. Furthermore, the steady state of maximal temperature during INM trials maintains the induced effect of the prior heating-up phase.
- The heating-up phase has a stronger impact on the negative amplitude of IEDs in $\mu$ECoG signals compared with the steady-state maximum temperature. This characteristic can be mitigated or its changes can be moderated using continuous-wave and pulsed NIR illumination.
- The ratio of delta and theta (2–8 Hz) band power to the total band (2–300 Hz) power shows a proportional increase during the heating-up phase, particularly when pulsed NIR light is used. However, during the steady-state maximum temperature, the relative power of delta and theta bands decreases proportionally.
- The increase in the ratio of gamma (28–80 Hz) band power is entirely correlated with increasing cortical temperature.
- While in the immediate vicinity of the stimulation site there are no clear changes in IED frequency associated with temperature. Additionally, steady-state maximum temperature during trials does not illustrate any consistent changes in IED frequency.
- The effects of INM on IED frequency and peak-to-peak amplitude exhibit great variability between animals. These effects are reversed depending on the frequency of pulsed-IR illumination.
- This effect of INM could potentially restrict its use in therapeutic applications related to epilepsy. However, the thermal effect of INM on cortical neurons induces changes in other characteristics of IED, which could prove beneficial for future applications.

Finally, the research outcomes highlight the following new hypotheses:

- There is a distinct effect of the heating-up phase and maximal cortical temperature on IED characteristics.
- There is reverse relation between the amplitude and frequency of IEDs and based on the frequency of NIR light.
- A low rate of heating up the cortex has a more inhibitory impact on the sharp negative-polarity of IEDs.
- There is no clear evidence that the INM procedure can suppress the IED frequency in anesthetized rodents.

### Supplementary Information

Supplementary Figures.

Supplementary Figures.

## Data Availability

The datasets generated and/or analyzed during the current study are available from the corresponding author upon reasonable request.
