# Author Correction: Thermal neuromodulation using pulsed and continuous infrared illumination in a penicillin-induced acute epilepsy model

**DOI:** 10.1038/s41598-023-45561-x

**Published:** 2023-11-02

**Authors:** Ebrahim Ismaiel, Richárd Fiáth, Ágnes Szabó, Ágoston Csaba Horváth, Zoltán Fekete

**Affiliations:** 1 Research Group for Implantable Microsystems, Faculty of Information Technology and Bionics, Pázmány Péter Catholic University, Budapest, Hungary; 2 Research Centre for Natural Sciences, Institute of Cognitive Neuroscience and Psychology, Magyar tudósok körútja 2, Budapest, 1117 Hungary; 3 Integrative Neuroscience Research Group, Faculty of Information Technology and Bionics, Pazmany Peter Catholic University, Budapest, Hungary

Correction to: *Scientific Reports* 10.1038/s41598-023-41552-0, published online 02 September 2023

The original version of this Article omitted an affiliation for Richárd Fiáth. The correct affiliations are listed below.

Research Centre for Natural Sciences, Institute of Cognitive Neuroscience and Psychology, Magyar tudósok körútja 2, Budapest 1117, Hungary.

Integrative Neuroscience Research Group, Faculty of Information Technology and Bionics, Pazmany Peter Catholic University, Budapest, Hungary.

The original Article has been corrected.

