# Supplementary material for: Thermal neuromodulation using pulsed and continuous infrared illumination in a penicillin-induced acute epilepsy model
Source: Sci Rep. 2023 Sep 2;13:14460. doi: 10.1038/s41598-023-41552-0 (PMC10475096; doi:10.1038/s41598-023-41552-0)
Supplement: Supplementary file 2 — Supplementary Figures. [file 41598_2023_41552_MOESM2_ESM.pdf]

Figure (A1.1). The last 2-sec of four ECoG sites (#1 refers to "beside optrode" site and #4 refers to "far from optrode" site). The figure contains five sub-figures where each sub-figure shows the ECoG of the three phases of INM (last 2-sec of 2-min-CTRL [PCtrl], 2-min-IR-ON [P2ON] and 4-min-IR-OFF [P4OFF]) during different pulsed frequencies of IR light and two trials of INM. Each channel or site (6 rows) expresses ECoG signals of 6 rats using ISP1 protocol. The red colored spikes are detected IEDs.

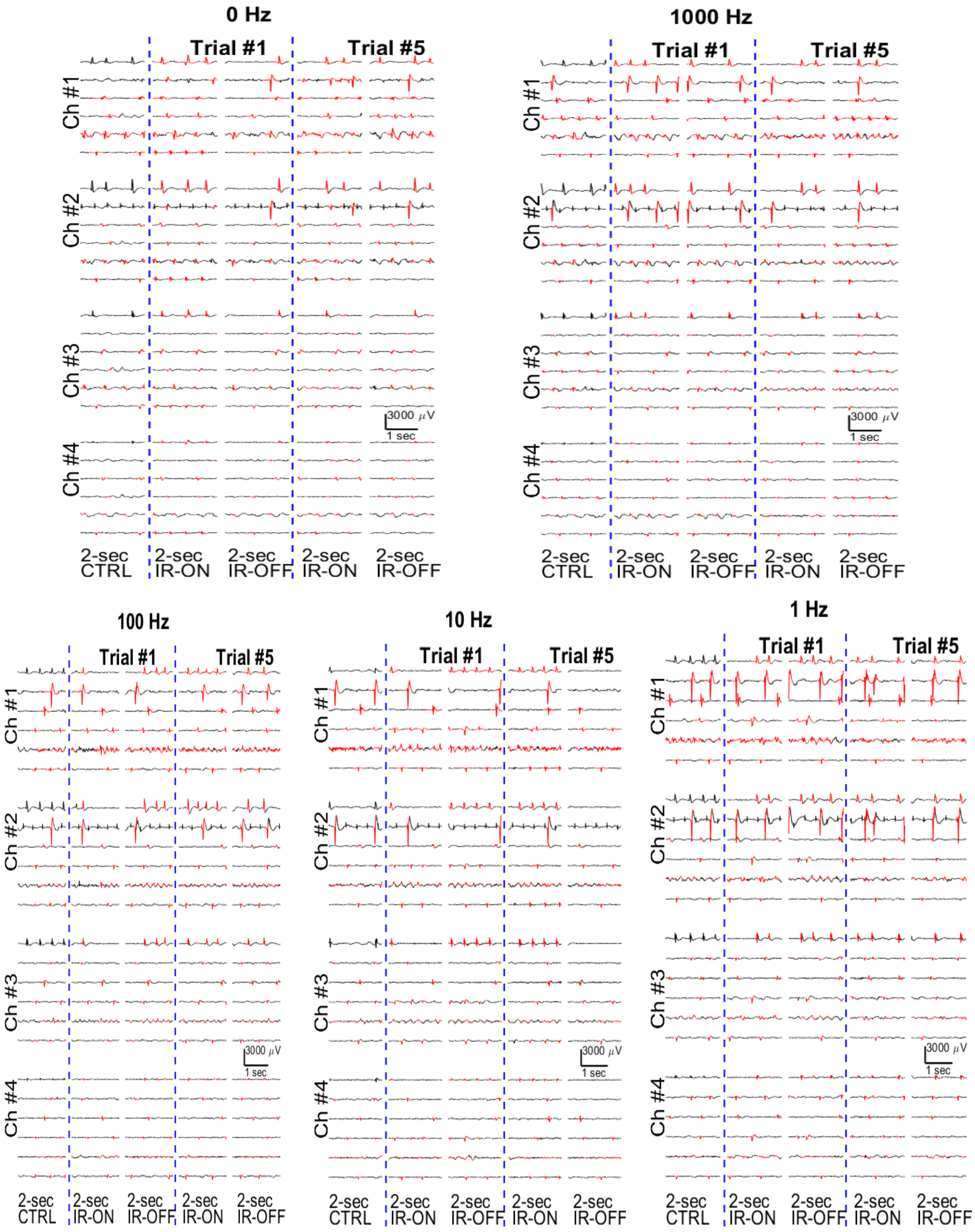

Figure (A1.2). Extracted peak-to-peak amplitude feature from detected IEDs in ECoG sites. For each pulsed frequency of IR light, there is a row shows the normalized change of peak-to-peak amplitude feature during the three main phases of INM with ISP1 protocol (phases are separated with dashed red line).

Peak-to-peak amplitude of detected IEDs				
Freq.	Ch#1 (Beside Optrode)	Ch#2	Ch#3	Ch#4 (Far from Optrode)
Continuous pulsed IR	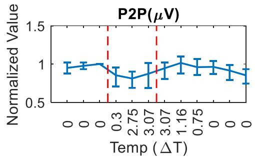	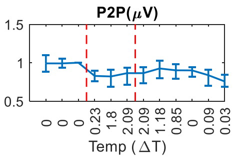	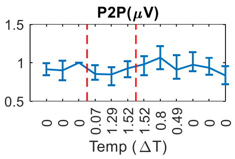	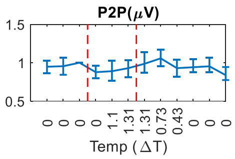
1000 Hz pulsed IR	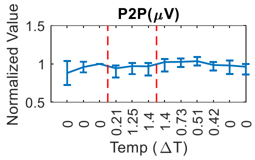	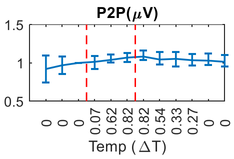	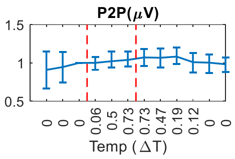	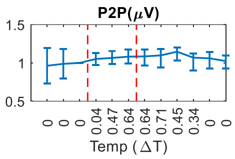
100 Hz pulsed IR	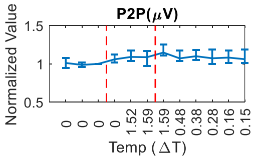	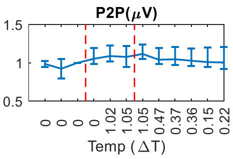	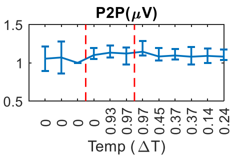	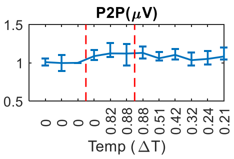
10 Hz pulsed IR	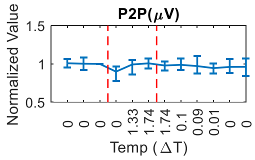	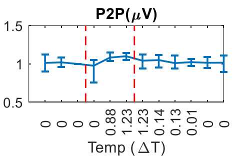	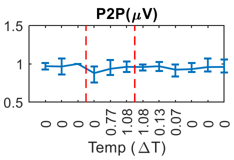	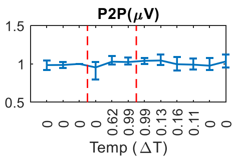
1 Hz pulsed IR	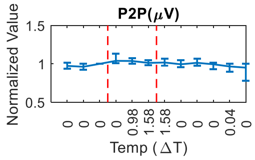	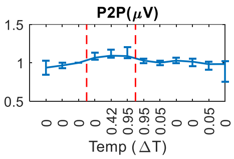	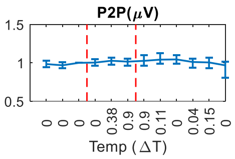	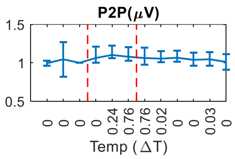

Figure (A1.3). Extracted negative amplitude feature from detected IEDs in ECoG sites. For each pulsed frequency of IR light, there is a row shows the normalized change of negative amplitude feature during the three main phases of INM with ISP1 protocol (phases are separated with dashed red line).

Negative peak to P2P ratio of detected IEDs				
Freq.	Ch#1 (Beside Optrode)	Ch#2	Ch#3	Ch#4 (Far from Optrode)
Continuous pulsed IR	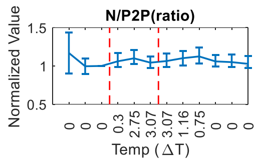	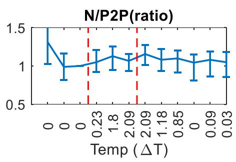	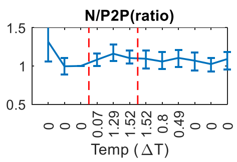	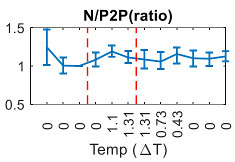
1000 Hz pulsed IR	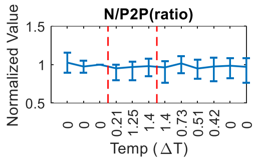	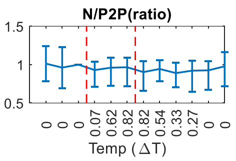	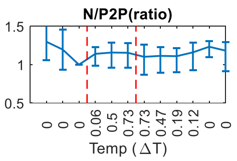	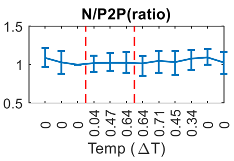
100 Hz pulsed IR	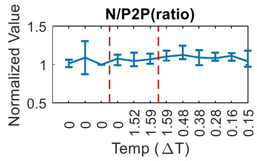	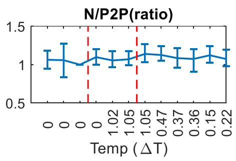	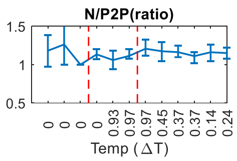	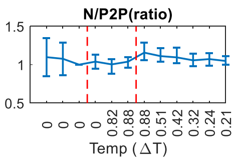
10 Hz pulsed IR	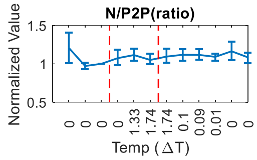	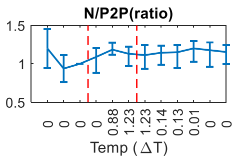	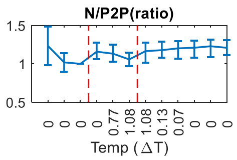	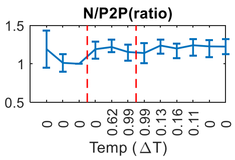
1 Hz pulsed IR	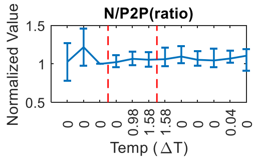	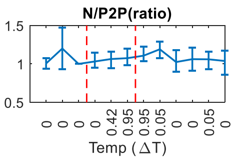	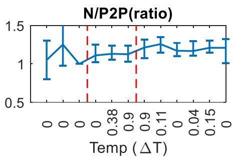	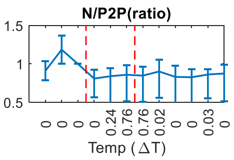

Figure (A1.4). Extracted ratio of 2-8 Hz band power from detected IEDs in ECoG sites. For each pulsed frequency of IR light, there is a row shows the normalized change of 2-8 Hz band power during the three main phases of INM with ISP1 protocol (phases are separated with dashed red line).

<table>
<tr><th colspan="5">(2-8 Hz) ratio of detected IEDs</th></tr>
<tr><th>Freq.</th><th>Ch#1 (Beside Optrode)</th><th>Ch#2</th><th>Ch#3</th><th>Ch#4 (Far from Optrode)</th></tr>
<tr><td>Continuous pulsed IR</td>
<td>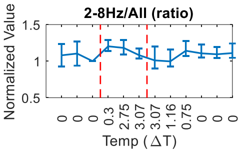
</td>
<td>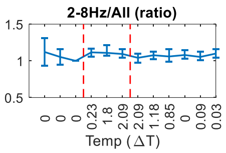
</td>
<td>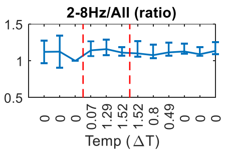
</td>
<td>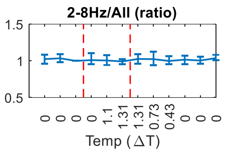
</td></tr>
<tr><td>1000 Hz pulsed IR</td>
<td>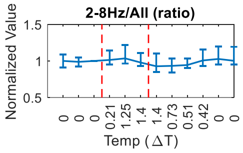
</td>
<td>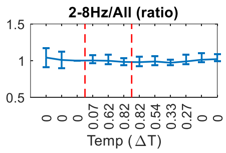
</td>
<td>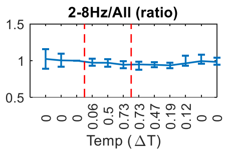
</td>
<td>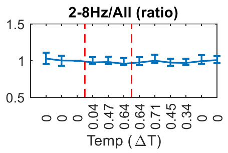
</td></tr>
<tr><td>100 Hz pulsed IR</td>
<td>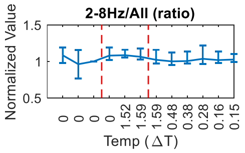
</td>
<td>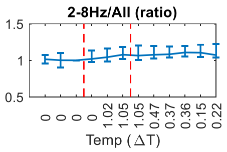
</td>
<td>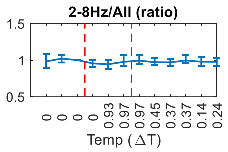
</td>
<td>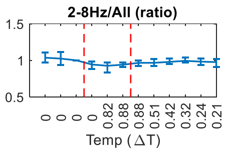
</td></tr>
<tr><td>10 Hz pulsed IR</td>
<td>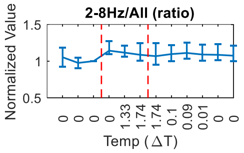
</td>
<td>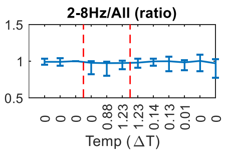
</td>
<td>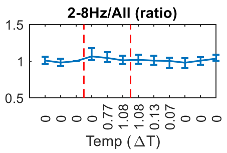
</td>
<td>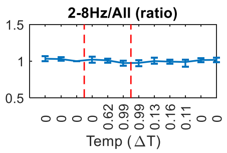
</td></tr>
<tr><td>1 Hz pulsed IR</td>
<td>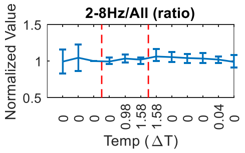
</td>
<td>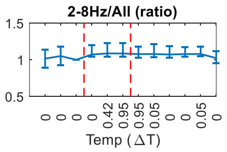
</td>
<td>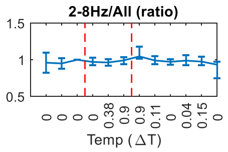
</td>
<td>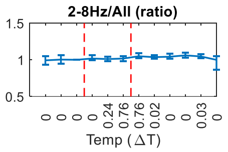
</td></tr>
</table>

Figure (A1.5). Extracted ratio of 28-80 Hz band power from detected IEDs in ECoG sites. For each pulsed frequency of IR light, there is a row shows the normalized change of 28-80 Hz band power during the three main phases of INM with ISP1 protocol (phases are separated with dashed red line).

(28-80 Hz) ratio of detected IEDs				
Freq.	Ch#1 (Beside Optrode)	Ch#2	Ch#3	Ch#4 (Far from Optrode)
Continuous pulsed IR	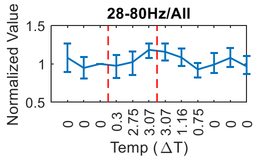	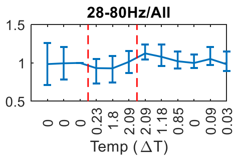	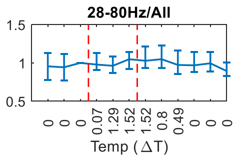	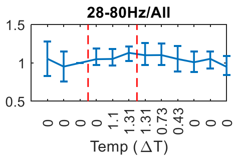
1000 Hz pulsed IR	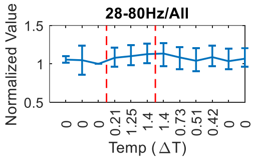	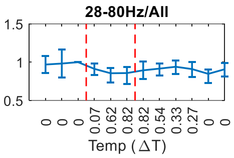	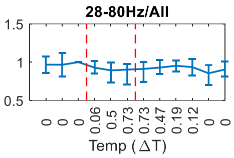	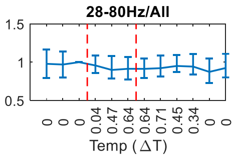
100 Hz pulsed IR	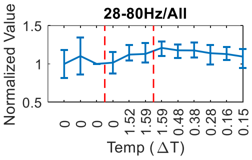	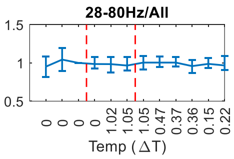	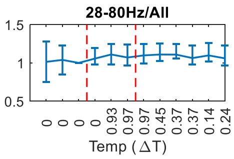	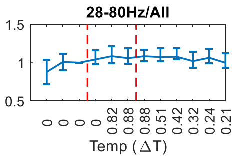
10 Hz pulsed IR	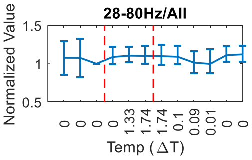	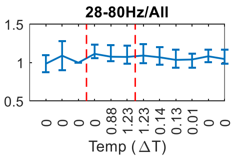	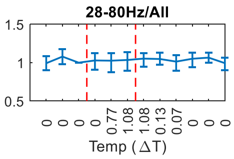	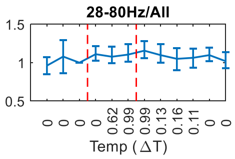
1 Hz pulsed IR	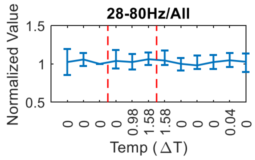	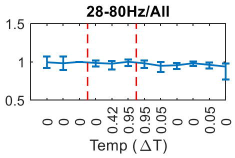	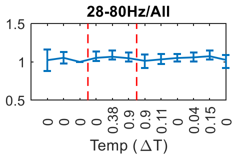	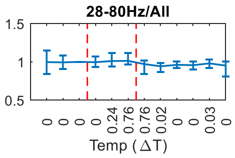

Figure (A1.6). Extracted IED frequency feature from detected IEDs in ECoG sites. For each pulsed frequency of IR light, there is a row shows the normalized change of IED frequency feature during the three main phases of INM with ISP1 protocol (phases are separated with dashed red line).

Spikes count of detected IEDs				
Freq.	Ch#1 (Beside Optrode)	Ch#2	Ch#3	Ch#4 (Far from Optrode)
Continuous pulsed IR	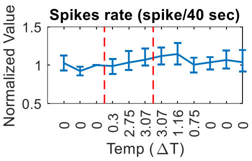	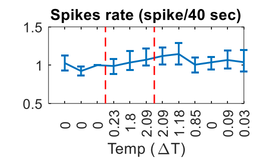	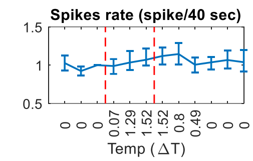	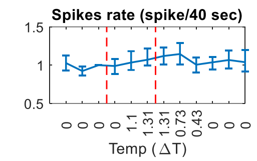
1000 Hz pulsed IR	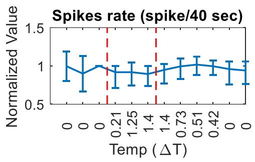	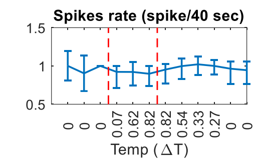	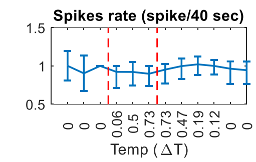	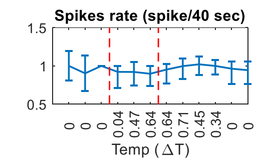
100 Hz pulsed IR	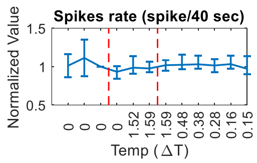	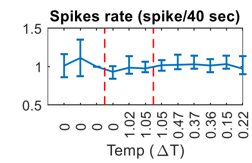	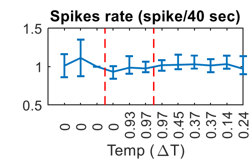	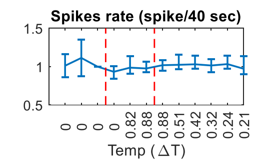
10 Hz pulsed IR	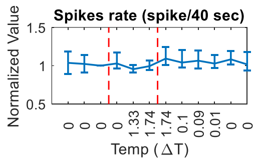	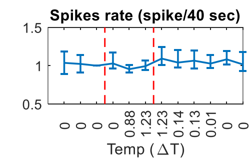	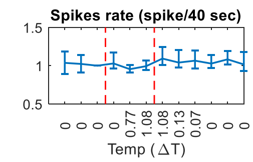	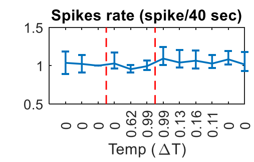
1 Hz pulsed IR	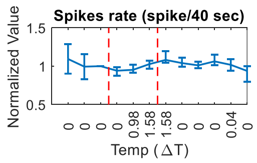	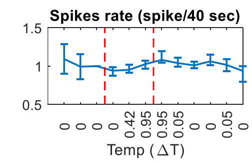	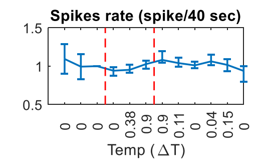	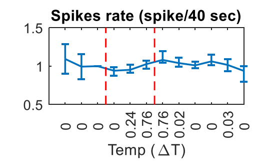
